# A cohort of women with ectopic pregnancy: challenges in diagnosis and management in a rural hospital in a low-income country

**DOI:** 10.1186/s12884-018-1777-2

**Published:** 2018-05-11

**Authors:** R. Mooij, G. C. Mgalega, I. H. Mwampagatwa, J. van Dillen, J. Stekelenburg

**Affiliations:** 1 Ndala Hospital, PO Box 15 Ndala, Tanzania; 2 VieCuri Medical Centre, Tegelseweg 210, 5912 BL Venlo, The Netherlands; 3 College of Health Sciences, University of Dodoma, PO Box 395 Dodoma, Tanzania; 4 Radboud University Medical Centre, Geert Grooteplein-Zuid 10, 6525 GA Nijmegen, The Netherlands; 5 Leeuwarden Medical Centre, Henri Dunantweg 2, 8934 AD Leeuwarden, The Netherlands; 6 University Medical Centre Groningen/University of Groningen, Antonius Deusinglaan 1, 9700 AD Groningen, The Netherlands

**Keywords:** Ectopic pregnancy, Abdominal aspiration, abdominocentesis, ultrasound, Diagnosis

## Abstract

**Background:**

Ectopic pregnancy (EP) is a serious complication of early pregnancy. In low-income countries diagnosis of EP is difficult and it is a major contributor to maternal mortality. We aimed to assess and improve the diagnostic process of women with EP.

**Methods:**

We conducted a retrospective medical records study of all women with confirmed EP in Ndala Hospital from 2010 to 2012. We used data on demographics, symptoms, diagnostic procedures, surgical findings, treatment and post-operative status.

**Results:**

Six thousand six hundred sixty-two women gave birth in the hospital, and 88 women were diagnosed with EP (incidence 1.3%). Thirty-nine percent of women did not report to be pregnant or to have a history of amenorrhea. On admission in Ndala hospital, a diagnosis of ‘suspected EP’ was made in less than half (47%) of the cases. Most women had a urine pregnancy test done (sensitivity of 98%). Peritoneal aspiration was done in 42%. The fifty-five women with EP who were diagnosed by ultrasound received a lower mean number of units of blood transfusion and had less often severe anaemia than women who were diagnosed by abdominal aspiration (abdominocentesis). The majority of women (65%) had surgery within 24 h after admission.

**Conclusions:**

Diagnosing EP in a rural hospital in Tanzania is challenging. Often there is a large doctors’ delay before the right diagnosis is made. Abdominal aspiration can be useful for rapid diagnosis. A pelvic ultrasound, when available, allows the diagnosis to be made earlier with less intra-abdominal bleeding.

## Background

Ectopic Pregnancy (EP) is a serious complication of early pregnancy. In low-income countries (LIC) it is a major contributor to maternal mortality, although exact incidence rates are unknown, due to frequent misdiagnosis [1, 2]. For the same reason case fatality rates are also not without bias, but reported between 1 and 3% [2]. In high-income countries, early diagnosis can often be made using ultrasound and serum human chorionic gonadotropin level [3].

In LIC, making the right diagnosis is more difficult, and delay in diagnosis can occur before and after consulting a healthcare worker [2]. The majority of deaths take place in the community or shortly after admission in a health institution, making EP a relevant public health issue [4]. Diagnosis is primarily made with history taking and findings of physical examination. Diagnostic tests like chorionic gonadotropin level, peritoneal aspiration and ultrasound are used if available [2, 5]. A negative urine pregnancy test can rule out EP. Peritoneal aspiration can confirm the presence of blood in the abdomen making the suspicion of EP very high. This can be done with culdocentesis or abdominal aspiration, but these tests are both not reliable when the EP is not ruptured [6]. This means that in LIC, especially when ultrasound is absent, diagnosis is most often made late (after rupture) [2, 5, 7]. When equipment and an experienced ultrasonographer are present in a low-income setting, pelvic ultrasound can help by early detection of free abdominal fluid of a leaking EP. Conversely, detection of an intra-uterine pregnancy makes EP unlikely. Ultrasound is associated with earlier diagnosis in some studies in tertiary centres in LIC and has been recommended for broader use [8–10]. It is shown to be feasible in district-hospitals as well [11].

In this study, we reviewed cases of women diagnosed with EP in a rural hospital in a LIC, and assessed the value of diagnostic tests including pelvic ultrasound, with the aim to upscale capacity if proven useful.

## Methods

### Setting

This study was done at Ndala Hospital, a private Catholic hospital, situated in the Tabora region, in a rural part of Western Tanzania. It serves a catchment area of around 200,000 people. Annually, there were roughly 2200 deliveries in the hospital. There was a poor referral infrastructure and many women were self-referrals from home or referred from health centres, rarely with any written handover. Some of these centres provided basic services for obstetric care and dilatation and curettage (D&C) to evacuate retained products of pregnancy.

Diagnostic modalities for EP included urine pregnancy test and ultrasound (Corevision Pro, SSA-350A, Toshiba Medical Systems, including vaginal probe), although there was not always a clinician available who was trained to perform (pelvic) ultrasound. Confirmation of haemoperitoneum was usually done through abdominal aspiration. Comprehensive Emergency Obstetric and Neonatal Care was available in the hospital and was conducted by four healthcare workers: one medical officer (medical degree) and three diploma-level assistant medical officers. Surgery (including D&C and laparotomy) was usually possible 24 h per day; there was no laparoscopy. Ndala Hospital had a blood bank, which was frequently without blood, so family members could donate whole blood after screening for Human Immunodeficiency Virus. There were virtually no possibilities for urgent referral and diagnostic and therapeutic options were similar in regional/referral hospitals.

### Participants

All patients with a surgically confirmed diagnosis of EP in Ndala Hospital from January 2010 to December 2012 were included and their data analysed. Admission and theatre logbooks were searched for the diagnosis, and the corresponding patient record was traced. Demographics, symptoms, diagnostic procedures, surgical findings, treatment and post-operative status were noted. The diagnosis of EP was made by inspection of the attending surgeon. Pathologic confirmation was not available.

### Statistical analysis

Data management was done using Microsoft® Office Excel® 2007, and statistical analysis was done with Epi Info™ 7 (Centers for Disease Control). *P*-values were calculated with Chi^2^ test and T-test where appropriate.

## Results

During the three year study period, 6662 women gave birth, and 88 women were diagnosed with EP (in-hospital incidence 1.3%). Eighty-nine women had suspected EP. Eighty-seven women had surgery at Ndala Hospital. Two women with suspected EP (positive urine pregnancy test and blood demonstrated intraperitoneally) were referred to another hospital because of a shortage of essential surgical supplies. Of one of those two, the outcome is known: surgery and a confirmed diagnosis. She was included in this study, making the total number of patients 88. The other woman was lost to follow up and was not included. During the study period, two women were recorded to have had surgery because of suspected EP but did not have EP. One had a miscarriage: the other had an early intrauterine pregnancy. From 85 patients (97%) the medical records were found. Characteristics of the included women are shown in Table 1.

**Table 1:** characteristics of patients with ectopic pregnancy

		n
Characteristics		
Mean age (years, standard deviation)	30.9 (6.7)	88
Median gravidity (interquartile range)	4 (3–7)	64
Mean length of admission (days, interquartile range)	6 (4–8)	88
Mean amenorrhea (months, standard deviation)	1.5 (1.2)	85
Women without amenorrhea / reported pregnancy (%)	33 (39)	85
Women with previous contact health care provider before admission, without diagnosis made (%)	60 (71)	85
Presenting symptoms		
Abdominal pain (%)	83 (98)	85
Vaginal bleeding (%)	51 (60)	84
Findings on admission		
Pelvic mass (%)	17 (20)	85
Abdominal distension (%)	33 (38)	85
Ectopic pregnancy suspected by attending health care worker at time of admission (%)	42 (47)	85

Thirty-three women (39%) did not report to be pregnant or to have amenorrhea. Three women (3%) had a history of an earlier EP. Two (2%) had previous bilateral tubal ligation. Almost all women complained of abdominal pain; only 60% of vaginal bleeding. Nearly 80% (60/85) of women had a recent previous contact with a healthcare provider because of abdominal pain or irregular bleeding. Eleven of them (11/60 = 18%) were treated as an outpatient in Ndala hospital, the majority (82%) at a traditional healer or healthcare worker from another facility. Two (2/60, 3%) were treated in health centres with D&C for an incomplete miscarriage.

On admission in Ndala hospital, a diagnosis of ‘suspected EP’ was made in less than half (47%) of the cases by the attending clinician after taking a history and physical examination. The majority of women (65%) had surgery within 24 h after admission, almost one quarter (24%) within 24–48 h and the remainder after 48 h (four patients the 3rd day, two the 5th day and one after ten days of admission).

**Fig. 1:**
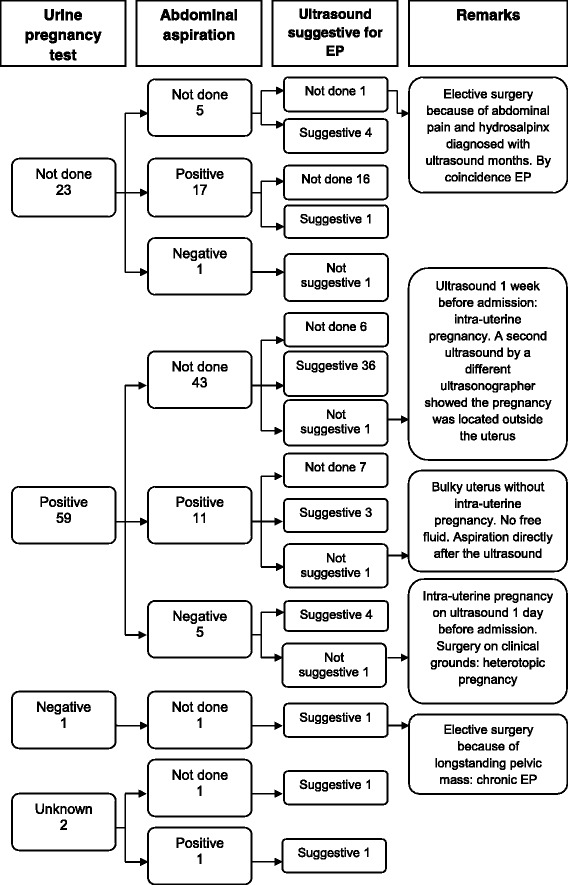
Diagnosis included women

Diagnostic procedures are shown in Fig. 1 and Table 2.

**Table 2:** diagnostic procedures

		N
Urine pregnancy test (%)		84
done – positive	60 (71)	
done – negative	1 (1)	
not done	23 (27)	
Mean haemoglobin count (g/dL, standard deviation)	8.2 (1.9)	80
Women with severe anaemia^a^ (%) (Hb < 7 g/dL)	20 (25)	
Abdominal aspiration (%)		86
done – positive	30 (35)	
done – negative	6 (7)	
not done	50 (58)	
Women with ultrasound (%)	55 (64)	86
Ultrasound findings		855
Not suggestive of ectopic pregnancy	3 (5%)	
Uterus without intra-uterine gestation	37 (67%)	
Free fluid cul du sac	34 (62%)	
Pelvic/adnexal mass	16 (29%)	
Extra-uterine gestational sac	6 (11%)	
Mean haemoglobin count (g/dL, standard deviation)	8.6 (2.0)	49
Ultrasound findings when aspiration negative		8
Free abdominal fluid without intra-uterine gestation	6	
Ectopic mass without intra-uterine gestation	1	
Not suggestive of ectopic pregnancy	1	

^a^WHO: Haemoglobin concentrations for the diagnosis of anaemia and assessment of severity. Vitamin and Mineral Nutrition Information System. Geneva: World Health Organization. 2011

Most women had a urine pregnancy test done, of which one was negative (sensitivity 98%). Peritoneal aspiration was done in 42% (36/86) and was found positive in 30 cases (83%). In the 19 cases where the patient had abdominal distension, aspiration was positive in 100%. In the 17 cases where aspiration was done in the absence of abdominal distension, it was significantly less often positive (11 patients, 65%). No complications of this procedure were recorded. In the six women with a negative aspiration, ultrasound was used for diagnosing EP in five cases. One woman with a negative aspiration was in hypovolaemic shock and had surgery on clinical grounds.

Fifty-five women underwent ultrasonography. Most often (65%) free fluid and an empty uterus were described; a pelvic or adnexal mass was seen in 30% of women. Three women (5%) had an ultrasound which was judged to be not suggestive for an EP (Fig. 1). These women with negative ultrasound had surgery on clinical grounds or because of changing condition after expectative management.

Location of the EP was Fallopian tube (82%), interstitial (4%) and ovarian (2%, Table 3).

**Table 3:** findings at surgery

		N
Side ectopic (%)		83
Left	29 (35)	
Right	52 (63)	
Bilateral	2 (2)	
Site ectopic (%)		83
Fallopian tube	68 (82)	
Interstitium	3 (4)	
Pouch of Douglas	3 (4)	
Ovary	2 (2)	
Mixed		
Fallopian tube and interstitium	2 (2)	
Fallopian tube and ovary	2 (2)	
Fimbrial end of Fallopian tube and pouch of Douglas	1 (1)	
Bilateral		
Fallopian tube bilaterally	2 (2)	
Adhesions (%)	24 (29)	83
Procedure (%)		84
Salpingectomy	67 (79)	
Salpingectomy and oophorectomy	5 (6)	
Salpingectomy and uterus repair	5 (6)	
Uterus repair	2 (2)	
Oophorectomy	2 (2)	
Subtotal hysterectomy	1 (1)	
Salpingotomy	1 (1)	
Removal ectopic tissue	1 (1)	
Mean blood loss (ml, standard deviation)	1234 (798)	73

In three women, the attending surgeon noted a double pregnancy: one heterotopic (intra-uterine and ectopic) and two bilateral EPs. Seven women (8%) underwent oophorectomy because of ovarian EP (2), tubal EP fixed to the ovary (2) and dense adhesions between EP and ovary (3). One woman underwent a subtotal hysterectomy because of an infected chronic EP with a frozen pelvis. Thirty-three women (40%) needed a blood transfusion of one or more units. Comparing women who were diagnosed EP with abdominal aspiration (*n* = 31) and women diagnosed EP by ultrasound only (*n* = 43), women diagnosed by ultrasound had a significantly lower mean number of units of blood transfusion and less often severe anaemia (15% vs. 30%, not statistically significant), see Table 4.

**Table 4:** outcome per diagnostic modality

Characteristic	Diagnosis made by positive aspiration	Diagnosis made by positive ultrasound	*P* -value
Mean amount of blood transfused (ml, standard deviation)	419 (485) (31)^a^	186 (378) (43)	0.01
Severe anaemia (Hb < 7 g/dL) (%)	9 (30) (30)	6 (15) (41)	0.20
Blood transfusion OR Hb < 7 g/dL (%)	20 (67) (30)	12 (30) (40)	0.05

^a^The number of patients is given on the second line between parenthesis

None of the women died. Serious complications occurred in 4 cases (5%). One woman had a bladder lesion which was detected pre-operatively and directly repaired and healed without any problem, and three women needed re-laparotomy due to suspected infection or bleeding. In two cases no abnormalities were found; in one woman a gauze was found that was accidentally left during the first surgery.

## Discussion

This retrospective case series study illustrates the diagnostic dilemmas with EP in a rural hospital in a low-income country. In these cases, multiple delay factors were identified. Phase 1 delay from the patient herself: 39% of women were not aware of being pregnant. Also, phase 3 delay was observed: 71% had previous contact with a formal or informal health care provider without the correct diagnosis being made, and 35% did not have surgery within 24 h after admission [12].

Abdominal pain was the only consistent finding, in 98% of included women. This supports the adage to always consider EP in all female patients of fertile age with abdominal pain.

Culdocentesis has long been an essential investigation for EP and still is the most important test in large parts of the world [13]. It has good diagnostic properties [14], even in the absence of classic peritoneal signs or signs of shock [15]. However, it is a difficult procedure for inexperienced clinicians, not without risk of complication [16, 17] and is replaced by pelvic ultrasound where available, which is less invasive and at least equally accurate because it can help diagnosing an EP before rupture [18].

In Ndala Hospital abdominal aspiration, or abdominocentesis, is commonly used when ultrasound is not directly available. There is little literature about the diagnostic value of this test or the risks [7, 19], and it is unknown if the procedure is commonly used, although abdominal aspiration (“four quadrant tap”) is advised in King’s standard textbook Primary Surgery for general doctors [5]. Abdominal aspiration was positive in all 19 patients with abdominal distension. This is the first study showing that this test is sensitive if used in women with ruptured EP and a distended abdomen. As EP mainly causes maternal mortality when there is a delay in diagnosis and treatment, a faster diagnosis can save lives [2, 4, 5, 20]. In case of a positive aspiration, omitting ultrasound reduces time (as well as costs) before surgery. Our study shows that for patients without abdominal distension or with a negative aspiration test, ultrasound is of added diagnostic value, with less need of blood transfusion, which is in line with other reports [10, 21]. When ultrasound is not available, depending on the clinical condition of the patient, diagnostic surgery can be considered.

**Fig. 2:**
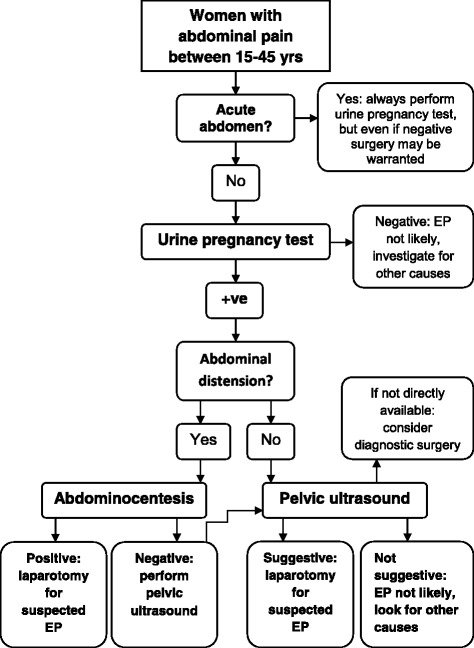
Diagnostic flowchart for women with possible EP

Based on the findings of this study we have constructed a diagnostic protocol for possible EP in our setting with limited availability of ultrasound (Fig. 2).

Training all clinicians to perform pelvic ultrasound could increase the availability of ultrasonography and reduce delay in diagnosis. Some clinicians have already been trained since the study period. Whether this improvement will lead to earlier diagnosis has to be reviewed in future studies.

Fortunately, in our series, no maternal deaths occurred. However, there was considerable morbidity as seven women needed oophorectomy and one had a hysterectomy. Late presentation, delay in diagnosis and limitations in the surgical skills of the general doctors might be determinants of this considerable morbidity.

### Study limitations

This is a retrospective study using routinely collected clinical records, and not all data could be reliably retrieved. Because of the dedicated research team and data collection shortly after discharge, most essential data is complete. This is a hospital-based study, only examining patients with confirmed EP. Within this design, it is impossible to determine the number of missed diagnosis. More importantly, we have no information about patients who did not reach the hospital. Since women who underwent ultrasound were often reviewed by an experienced clinician at the same time, it cannot be excluded that clinical judgment affected the results of the ultrasonography. Blood loss during surgery was not measured but estimated. However, no deaths occurred, and blood transfusions and haemoglobin counts were used as another way of establishing blood loss. Diagnosis of EP was made by the attending surgeon and was not histopathologically confirmed, but this is common in practice and studies in low-resource settings [15, 22].

## Conclusions

This study shows the diagnostic and therapeutic challenges of EP in a rural hospital in Tanzania. Often there is a large delay in reaching the right diagnosis and patients see different health care workers before the correct diagnosis is made. Abdominal aspiration can be useful for rapid diagnosis in patients with a distended abdomen and gross hematoperitoneum. Pelvic ultrasound, when available, allows the diagnosis to be made earlier before rupture or with less intra-abdominal bleeding, with the need for significantly less blood transfusion.

## Abbreviations

EP: Ectopic Pregnancy
LIC: Low-Income Country
